# Effective band gap engineering in multi-principal oxides (CeGdLa-Zr/Hf)O_x_ by temperature-induced oxygen vacancies

**DOI:** 10.1038/s41598-023-29477-0

**Published:** 2023-02-09

**Authors:** Yixuan Hu, Mariappan Anandkumar, Joydip Joardar, Xiaodong Wang, Atul Suresh Deshpande, Kolan Madhav Reddy

**Affiliations:** 1 State Key Laboratory for Metal Matrix Composites, School of Materials Science and Engineering, Shanghai Jiao Tong University, Shanghai, 200240 China; 2 Department of Materials Science and Metallurgical Engineering, Indian Institute of Technology Hyderabad, Sangareddy, 502285 India; 3 International Advanced Research Centre for Powder Metallurgy and New Materials (ARCI), Balapur P.O., Hyderabad, Telangana 500005 India

**Keywords:** Transmission electron microscopy, Photocatalysis, Computational methods

## Abstract

Oxygen vacancy control has been one of the most efficient methods to tune the physicochemical properties of conventional oxide materials. A new conceptual multi-principal oxide (MPO) is still lacking a control approach to introduce oxygen vacancies for tuning its inherent properties. Taking multi-principal rare earth-transition metal (CeGdLa-Zr/Hf) oxides as model systems, here we report temperature induced oxygen vacancy generation (OVG) phenomenon in MPOs. It is found that the OVG is strongly dependent on the composition of the MPOs showing different degrees of oxygen loss in (CeGdLaZr)O_x_ and (CeGdLaHf)O_x_ under identical high temperature annealing conditions. The results revealed that (CeGdLaZr)O_x_ remained stable single phase with a marginal decrease in the band gap of about 0.08 eV, whereas (CeGdLaHf)O_x_ contained two phases with similar crystal structure but different oxygen vacancy concentrations causing semiconductor-to-metal like transition. Due to the intrinsic high entropy, the metallic atoms sublattice in (CeGdLaHf)O_x_ remains rather stable, regardless of the interstitial oxygen atoms ranging from almost fully occupied (61.84 at%) to almost fully empty (8.73 at%) state in the respective crystal phases. Such highly tunable oxygen vacancies in (CeGdLa-Zr/Hf) oxides show a possible path for band gap engineering in MPOs for the development of efficient photocatalysts.

## Introduction

Oxygen vacancies have been the most common modulated defects in oxide materials for manipulating the physicochemical properties^1–3^. The presence of oxygen vacancies provides exposed active sites for catalysis reactions and alters the electronic structure, which can significantly improve the catalytic performance^2^. For instance, oxygen vacancies introduced into titanium oxide (TiO_2_) creates more active sites thus can harvest more visible light and enhance the selective photocatalytic CO_2_ reduction activity by up to 18 times^4,5^. It may be noted that oxygen vacancies^6^ were introduced in TiO_2_ by doping, annealing, and plasma treatment^6,7^. However, in the context of the emerging and promising MPO based catalysts^8,9^, it is crucial to find an applicable way to introduce oxygen vacancies.

Multi-principal oxides, formulated with the rapid development of multi-principal element alloys, have opened up an unprecedented large compositional space for alternative oxides catalysts. This new concept offered the opportunity to break the limitation of binary metal oxide catalysts that could not be overcome by traditional methods such as doping. At present, some MPOs have already been discovered as efficient catalysts^10–14^. For instance, dual-phase TiZrNbHfTaO_11_ exhibited high activity for CO_2_ conversion^13^, and TiZrHfNbTaO_x_ has been found to be efficient in photocatalytic hydrogen evolution^11^. However, these early attempts of MPOs on photocatalysts^10–14^ have been focused on synthesizing MPOs with various compositions and exploring their relative catalytic performance in the pristine states. Studies on MPOs in the defect state, especially the introduction of defects or the use of defects to modify physicochemical properties, are still limited, while defects such as oxygen vacancies have proven their importance in various binary oxides. The previous report on the preparation of MPO materials^15^ provided an indication that the regulation of composition and the oxygen vacancies introduced by the composition adjustment may be an effective strategy for controlled band gap engineering and catalytic performance. Moreover, the high configurational entropy in MPOs favors their higher ability to accommodate oxygen vacancies in a larger range than the binary oxides. It is envisaged that developing a facile route such as high-temperature annealing to introduce oxygen vacancies in MPO for band gap engineering is likely to bring about significant interest in the generation of more efficient MPO based catalysts.

In this work, we report the influence of high temperature annealing on the composition dependent oxygen vacancy generation in (CeGdLaZr)O_x_ (Zr-MPO) and (CeGdLaHf)O_x_ (Hf-MPO). A series of characterization including aberration-corrected (Cs) transmission electron microscopy (TEM), X-Ray Diffraction (XRD), Raman spectroscopy, differential scanning calorimetry (DSC), photoluminescence spectroscopy (PL) and theoretical calculation based on density functional theory (DFT) were employed for in-depth insight into the formation of oxygen vacancies and phase evolution in both the MPOs and their role on the band gap energy and photocatalysts.

## Results

### Structure and stability of Zr-MPO and Hf-MPO

**Figure 1:**
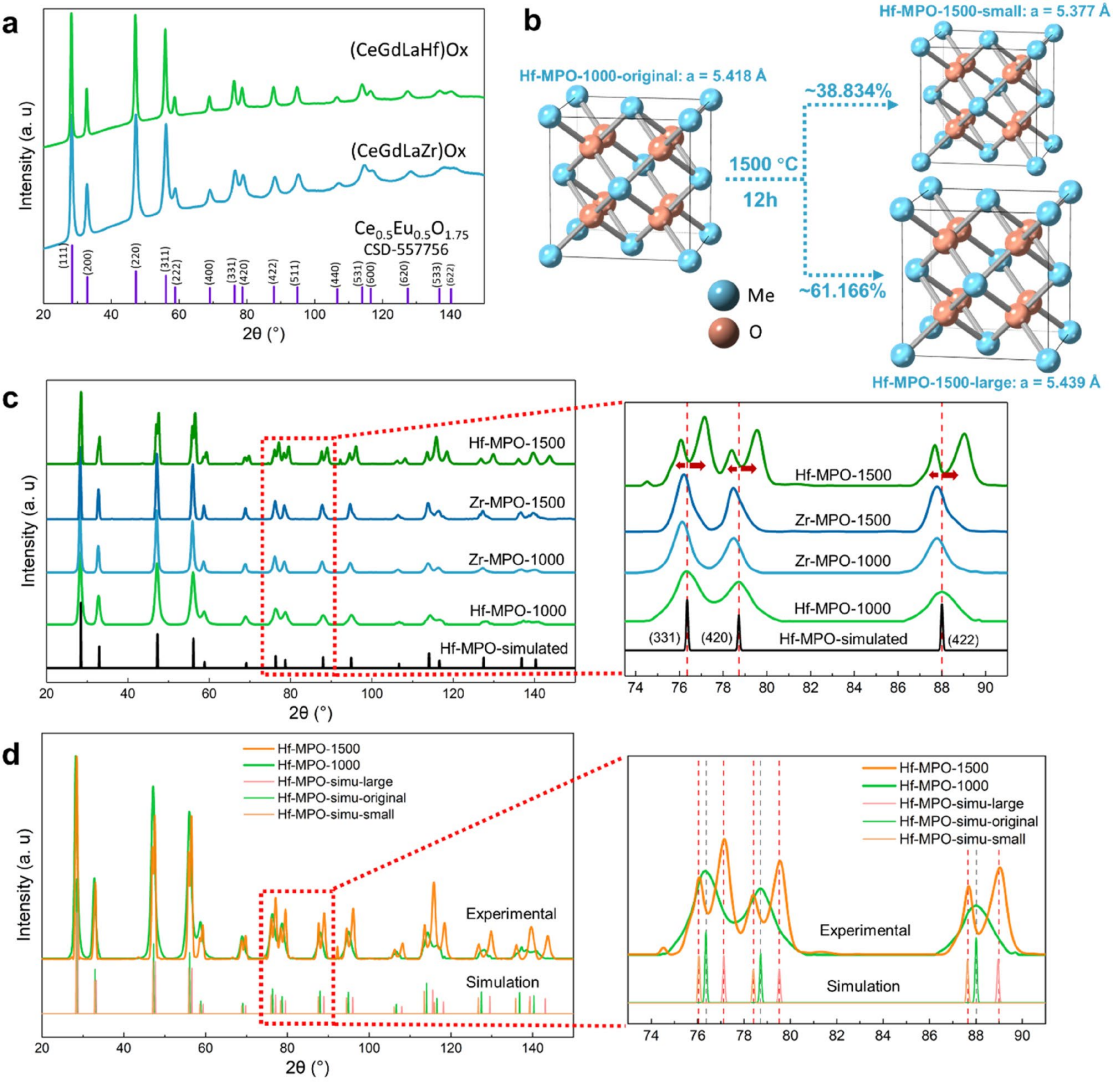
(**a**) XRD profiles of Zr/Hf-MPO annealed at 1000 °C. (**b**) Schematic illustrates phase separation in Hf-MPO when annealed at 1500 °C. Blue atoms Me represent Ce, Gd, La, Hf/Zr. (**c**) Experimental XRD profiles of Hf-MPO-1500, Zr-MPO-1500, Zr-MPO-1000, Hf-MPO-1000 overlaid with simulated Hf-MPO’s profile. (**d**) Comparison of the experimental XRD profiles of Hf-MPO-1500 and Hf-MPO-1000 with simulated XRD of the Hf-MPO-simu-small and Hf-MPO-simu-large and a single phase of Hf-MPO-simu-original composition.

Figure 1a shows the XRD profiles of the as-synthesized Zr-MPO and Hf-MPO and together overlaid with the standard peak positions of Ce_0.5_Eu_0.5_O_1.75_ (ICSD-557756) with cubic fluorite structure. It is evident that the peaks of the as-synthesized MPOs match well with Ce_0.5_Eu_0.5_O_1.75_. Moreover, the peak positions and relative intensities of both the MPOs are very close to each other (Fig. 1a), which suggests their similarities in crystal structures. Such similarities stem from their composition as the difference is only with one element, i.e., Hf vs. Zr. As Hf is next to Zr in the group (IV) metals of the periodic table, their binary oxides have very similar properties like structure, ionic radius, valence state, etc., as shown in Supplementary Table S1.

Figure 1c shows the XRD profiles of both MPOs after annealing at 1000 °C and 1500 °C. The XRD peaks of Zr-MPO-1000 and Hf-MPO-1000 are at the identical position to those of the as-synthesized MPOs and no other detectable secondary phase is observed. Despite the excellent thermal stability of Hf-MPO and Zr-MPO at 1000 °C, significant structural variation occurs when annealed at 1500 °C. Although the Zr-MPO-1500 peaks remain at the same position as Zr-MPO-1000, the Hf-MPO-1500 peaks split when compared with Hf-MPO-1000 (see the enlarged view of Fig. 1c). From Fig. 1d, it is more obvious to observe peaks splitting in Hf-MPO-1500 with one shifting to the lower angle (left) and another shifting to the higher angle (right) compared with those of Hf-MPO-1000. Such peak splitting is often related to the spinodal decomposition phenomenon^16^ in which two phases evolve with the same structure but differ in composition from that of the parent phase. Thus, it is reasonable to assume that two phases formed in Hf-MPO-1500 with same crystal structure but different compositions, as illustrated schematically in Fig. 1b. Therefore, the lattice constants for both types of MPOs were ascertained using Rietveld refinement method. Initially, the refinement is performed on the XRD patterns of Hf-MPO-1000, Zr-MPO-1000, Zr-MPO-1500 to optimize the lattice constant of the single cubic fluorite phase (shown in Supplementary Fig. S1a–c). The obtained lattice constant for Hf-MPO-1000 is 5.418 Å and the corresponding computed XRD profile is displayed as solid black lines in Fig. 1c. Further, we applied Rietveld refinement on Hf-MPO-1500 based on the ‘two phases’ assumption and the associated simulated phase’s profile named as Hf-MPO-simu-small and Hf-MPO-simu-large (Fig. 1d). The corresponding structure model of Hf-MPO-simu-small and Hf-MPO-simu-large constructed analogously to Hf-MPO-original composition (Fig. 1b) displaying four metallic cations that are randomly distributed in the FCC sublattice and anion (oxygen) atoms occupy the tetragonal interstitials preferentially. The results based on the ‘two phases’ assumption showed a low error function with Rb equals to 3.689% (see Fig. S1c) and the determined lattice constant for Hf-MPO-simu-small is 5.377 Å and Hf-MPO-simu-large is 5.439 Å. Accordingly, the calculated phase fractions of the Hf-MPO-1500-small is about ~ 61 wt% and Hf-MPO-1500-large is about ~ 39 wt%, respectively.

### Origin of phase separation in Hf-MPO at elevated temperature

**Figure 2:**
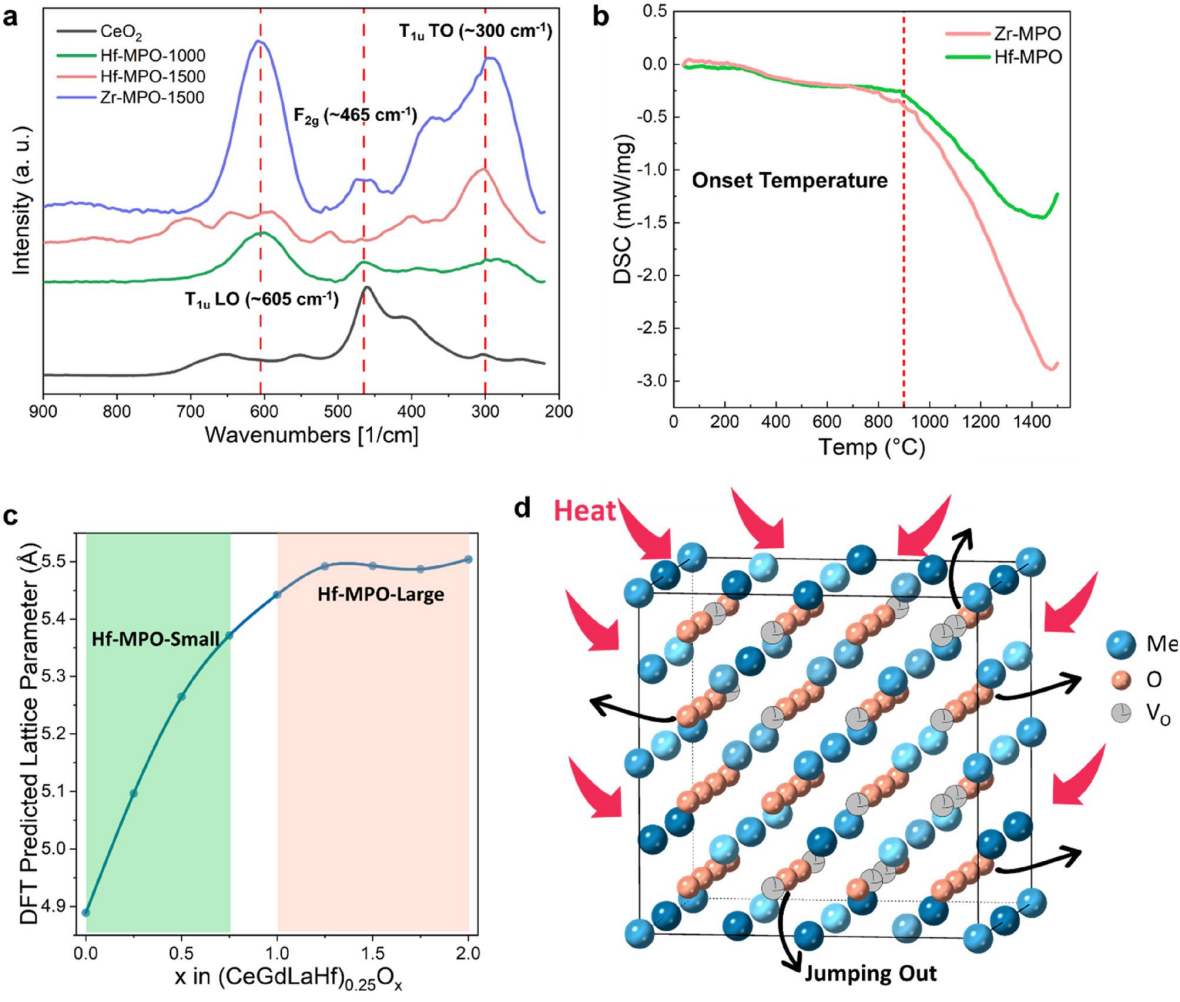
(**a**) Raman spectroscopy of Zr-MPO-1500, Hf-MPO-1500, Hf-MPO-1000, and standard fluorite CeO_2_). (**b**) DSC curve of Zr-MPO and Hf-MPO. (**c**) Predicted relationship between oxygen contents x of (CeGdLaHf)O_x_ and lattice constants based on the DFT study. (**d**) Schematic description of the oxygen liberation process for MPOs during annealing.

To understand the intrinsic reason for phase separation in Hf-MPO when compared with Zr-MPO at identical elevated temperature conditions, we have employed Raman spectroscopy and DSC studies along with DFT calculations. Figure 2a presents a comparison between the Raman spectra of MPOs, except for the Zr-MPO-1000, as it shows no difference in vibration modes with Hf-MPO-1000. Based on group theory analysis, fluorite structure has six optical-phonon branches, resulting in 3 different zone-center frequencies viz. triply degenerate Raman-active mode (F_2g_ ~ 465 cm^−1^), doubly degenerate TO mode (T_1u_ TO ~ 300 cm^−1^) and non-degenerate LO mode (T_1u_LO ~ 605 cm^−1^)^15,17,18^. Among these only F_2g_ mode appears in the standard CeO_2_ spectrum (Fig. 2a) as it represents the eightfold Ce–O bond vibration in fluorite structure. This characteristic degrades with the appearance of oxygen vacancies, which is to compensate for the charge imbalance brought by the presence of heterovalent cations such as La^3+^ and Gd^3+^ in MPOs^15^. Thus, the symmetry breaks with the degradation of peaks at around F_2g_ (~ 465 cm^−1^) as can be viewed from the spectra of Zr-MPO-1500, Hf-MPO-1000, and Hf-MPO-1500. Additionally, the existence of oxygen vacancies in these MPOs not only decreases the peak intensity of F_2g_ but also increases the peak intensity of T_1u_LO mode, which is consistent with prior literature^15,17^. Thus, the oxygen vacancies difference can be obtained by comparing the peak intensity and shape of T_1u_ LO mode. For MPOs annealed at 1000 °C, spectra show a small peak at 605 cm^−1^, which represents the necessary oxygen vacancies for charge compensation. But when MPOs annealed at 1500 °C, it shows different features regarding the T_1u_LO mode. The more intense peak at 605 cm^−1^ in Zr-MPO-1500 indicates an excess formation of oxygen vacancies at a higher temperature. While in Hf-MPO-1500, the peak decays and splits into several sub-peaks compared to Hf-MPO-1000, which implies multiple phases with varying oxygen concentrations. These results are in line with the XRD data (Fig. 1), where the observed peak splitting in Hf-MPO-1500 indicates the presence of two different Hf-MPO phases. Moreover, the difference in Raman vibration modes of Hf-MPO-1500 and Zr-MPO-1500 demonstrates that the reaction kinetics between 1000 and 1500 °C is highly composition-dependent.

In order to track the heat flow and structural transition associated kinetics of MPOs, the differential scanning calorimetry (DSC) was performed in the range of 50–1500 °C temperature. The DSC plots in Fig. 2b show an endothermic peak at the onset temperature of 900 °C for both Zr-MPO and Hf-MPO revealing that their crystal structure remains thermally stable up to this annealing temperature. However, the endothermic peak starts to vary significantly from 1000 to 1500 °C, indicating that there is a difference in the thermal stability and structural conformation between Zr-MPO and Hf-MPO. This broad endothermic peak has a shape similar to that observed in the oxygen loss process in vanadium pentoxide^19^, which indicated the possible occurrence of oxygen loss in Zr/Hf-MPO above 1000 °C. As Raman measurements (see Fig. 2a) pointed out excessive oxygen vacancies in Zr-MPO-1500 when compared with Zr-MPO-1000, it can be deduced that the oxygen vacancy generation (OVG) process is responsible for the observed endothermic peak in DSC. Considering the similar endothermic peak shape and chemical similarities of Zr-MPO and Hf-MPO, one would expect OVG in Hf-MPO as well. Moreover, this evidence shows that the elevated ambient temperature offers enough energy for the metal–oxygen bonds to dissociate and expand the lattice of the MPOs, similar to vanadium pentoxide^19,20^, which leads to the liberation of atoms especially fast migration of oxygen from tetrahedral interstitial sites to create vacancies as illustrated in Fig. 2d. On further decrease in temperature, the lattice shrunk and inhibits oxygen atoms to re-enter MPO lattice due to sluggish diffusion^21^, resulting in the formation of extra oxygen vacancies, which has been confirmed by Raman measurements at room temperature (see Fig. 2a). From DSC curve (Fig. 2b), it can be inferred that the less associated heat flow in Zr-MPO at elevated temperature makes the structure more stable when compared to Hf-MPO at the same condition. Therefore, the result confirms that the extent of OVG in Hf-MPO-1500 is greater than in Zr-MPO-1500, but how the OVG causes the phase separation with two varied lattice parameters in Hf-MPO is still not clear.

Thus, we applied DFT calculations to study the relation between lattice constant and oxygen concentration in Hf-MPO. Due to the impracticality of exploring the whole composition space of (CeGdLaHf)O_x_ Hf-MPO) systems, we determined lattice constant-oxygen contents relation for (CeGdLaHf)_0.25_O_x_ (x = 0.0, 0.25,…2.0) with equimolar metallic cations. To minimize the influence of supercell size and chemical disorder in different simulation cells, we performed calculations based on multiple supercells and showed the averaged results in Fig. 2c. It is revealed that with the increase in oxygen concentration from x = 0.0 to $\mathrm{x}$=2.0, the lattice parameter of Hf-MPO shows an increasing trend. The accuracy of the theoretically predicted trend can be verified by comparing it with Hf-MPO-1000, in which the OVG process didn’t take place (Fig. 2b). The refined lattice constant of Hf-MPO-1000 is 5.43172 Å and the corresponding averaged oxygen concentration is about 61.84% (clarified in latter section), this falls near the point of x = 1.75 on the curve, where predicted lattice constant is 5.48725 Å. Although the composition of Hf-MPO-1000 deviated from the composition we adopted in the DFT study, the minor difference in the lattice constants (1.02%) can still be an indicator to prove the accuracy of the DFT-predicted lattice constants. Thus, based on the relationship of predicted lattice constants versus oxygen contents, it becomes clear that the two phases with similar fluorite structures but different lattice constants found in Hf-MPO-1500 are Hf-MPOs with different oxygen contents. Consequently, it is possible to determine the oxygen contents range for the two separated phases in Hf-MPO-1500 with the refined lattice constants. The obtained refined lattice constant for Hf-MPO-1500-large is 5.45010 Å giving an approximate oxygen content in the range of 1.0 ≤ x ≤ 2.0, whereas for Hf-MPO-1500-small is 5.38572 Å giving an approximate oxygen content in the range of $\mathrm{x}$ ≤ 0.75. However, it should be noticed here that the lattice constants of Hf-MPO-1500-small and Hf-MPO-1500-large are of very limited difference 0.06528 Å, which makes accurately distinguishing the two phases theoretically difficult when considering the error bar as 5%. Therefore, it is important to recognize from DFT prediction that the phase separation in Hf-MPO-1500 is caused by two different oxygen concentrations, which come from the extent of OVG during annealing.

### Atomic scale analysis and phase separation understanding in Hf-MPO

**Figure 3:**
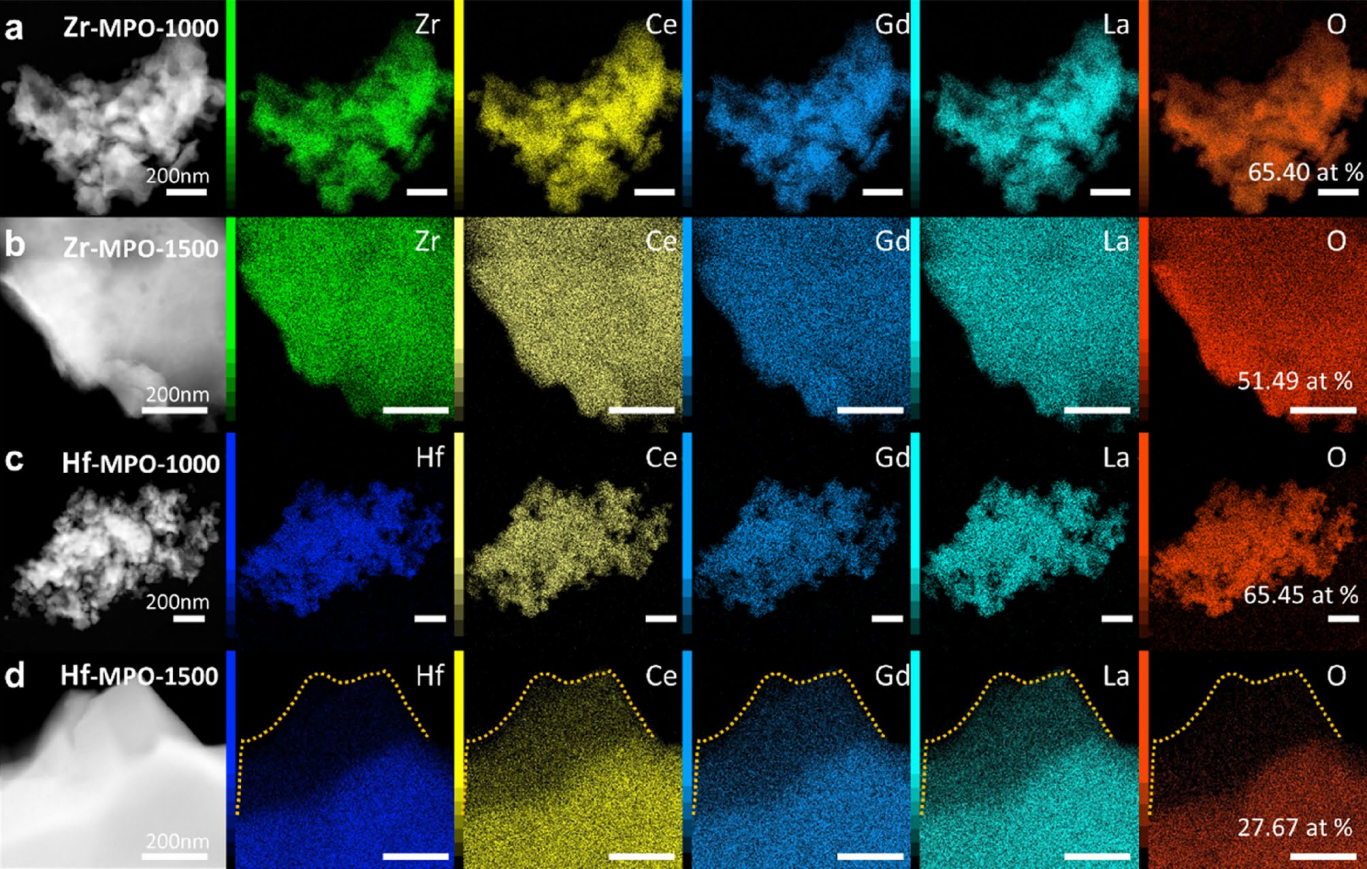
Typical STEM-EDS result of (**a**) Zr-MPO-1000; (**b**) Zr-MPO-1500; (**c**) Hf-MPO-1000 and (**d**) Hf-MPO-1500. The scale bar is 200 nm.

As mentioned before, composition differences in MPOs affect the OVG process greatly and further result in the phase separation in Hf-MPO. Thus, we have investigated the elemental composition and distribution in Hf-MPO and Zr-MPO annealed samples using analytical aberration-corrected scanning TEM (STEM). The representative STEM-Energy Dispersive Spectroscopy (STEM-EDS) result of Hf-MPO and Zr-MPO annealed samples are shown in Fig. 3. The measured elements composition of oxygen and metal cations from the individual grains are shown in the plot of Supplementary Fig. S3. The average oxygen concentration of Hf-MPO-1000 and Zr-MPO-1000 are 61.84 at% and 63.11 at%, respectively. Compared with the charge compensated oxygen content 63.64 at% in Hf/Zr-MPO with an equal cation ratio, neither Hf-MPO-1000 nor Zr-MPO-1000 exhibit obvious oxygen loss, which is in line with the DSC results, where the OVG starts from ~ 900 °C, making it insufficient to generate oxygen vacancies at 1000 °C. Thus, Hf-MPO-1000 and Zr-MPO-1000 remained in a single phase with insignificant loss of oxygen concentration. While annealing at 1500 °C, the OVG becomes intense and leads to significant oxygen loss in both Hf-MPO and Zr-MPO. STEM-EDS maps of Zr-MPO-1500 display uniform oxygen and cations similar to Zr-MPO-1000 (see Fig. 3a,b). The measured oxygen concentration in the Zr-MPO-1500 is about 50.25 at. % (i.e., x =  ~ 1.25), which is 21% lower than Zr-MPO-1000. In contrast, the grains observed in Hf-MPO-1500 can be classified into ‘oxygen-poor’ and ‘oxygen-rich’ regions with respect to the oxygen concentration. As indicated by the DFT prediction (Fig. 2c), the lattice constants vary significantly only when the oxygen content is in the range of x ≤ 0.75 and x ≥ 1.25. The average calculated value containing 15.43 at% oxygen from the ‘oxygen-poor’ region can be assigned to the Hf-MPO-1500-small phase, whereas 55.66 at% oxygen from the ‘oxygen-rich’ region can be assigned to the Hf-MPO-1500-large. Moreover, in the case of Hf-MPO, Hf tends to aggregate with O, and this feature is more obviously evident when temperature increases from 1000 to 1500 °C. This local oxygen-rich with Hf aggregation in Hf-MPO-1000 (Fig. 3c) confirms the influence of the local bonding environment on OVG in MPOs. With increasing temperature, the OVG is enhanced and shows a significant difference between the Hf segregated and Hf non-segregated regions. It can be observed in Hf-MPO-1500 (Fig. 3d) the local oxygen concentration became poor when Hf dropped sharply. The high affinity between Hf and O (the DFT calculated affinities of different metallic elements are listed in Supplementary Table S2) makes Hf diffuse to region’s rich with oxygen, and high temperature further eased the process. At the same time, as Hf keeps diffusing away, the regions lack of Hf endures more oxygen loss. This process continues to result in oxygen-rich and oxygen-poor regions, which led to phase separation on a macroscopic scale as identified in Hf-MPO-1500.

**Figure 4:**
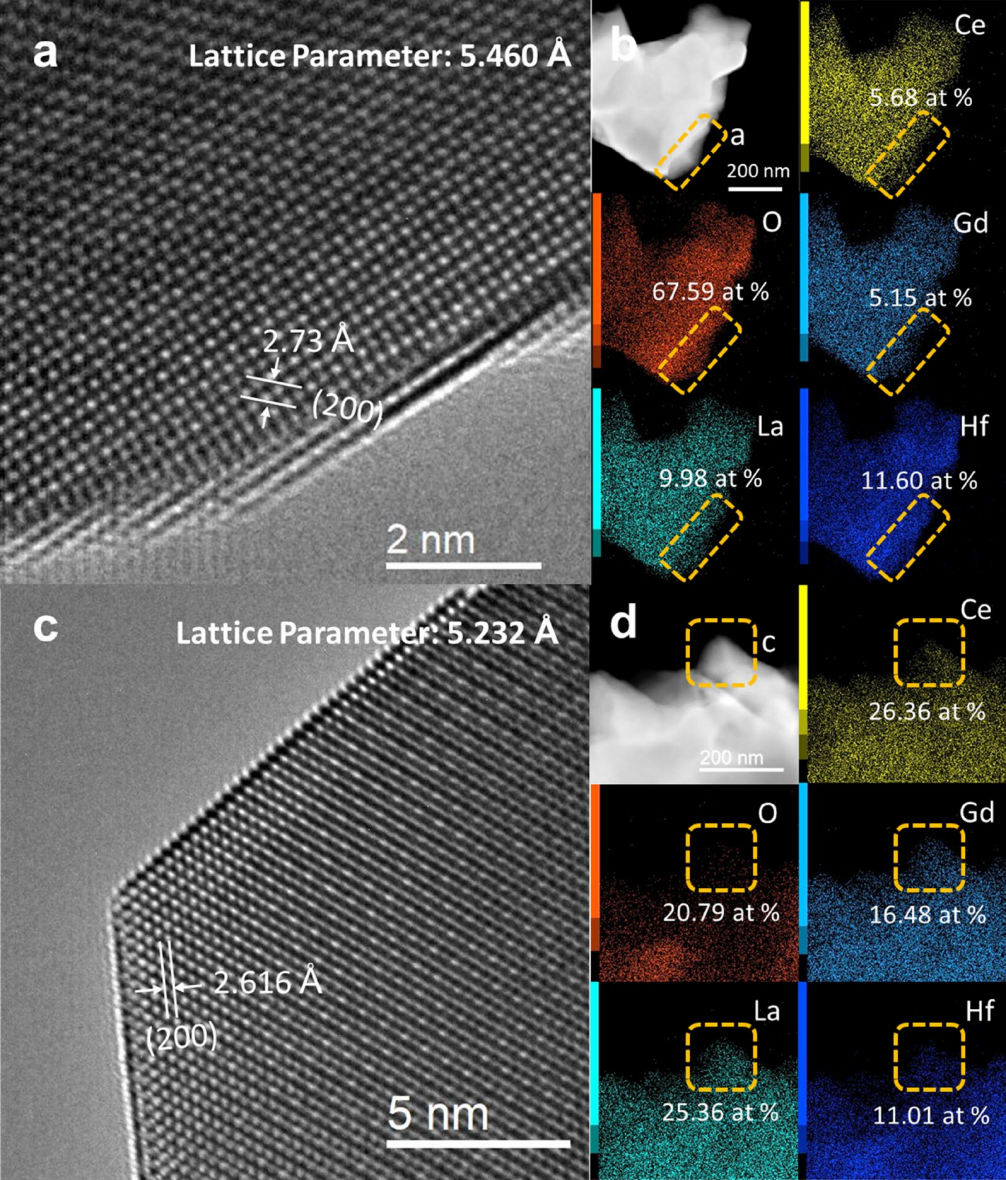
HRTEM image of (**a**) Hf-MPO-1500-Large and (**c**) Hf-MPO-1500-Small. STEM-EDS results: (**b**) corresponds to (**a**); (**d**) corresponds to (**c**). The labeled concentration in STEM-EDS results belong to the region circled by yellow dotted box.

The Hf-MPO-1500 with two different oxygen concentrations was further examined by HRTEM and STEM-EDS at the nanoscale. HRTEM image (Fig. 4a) taken along the [100] direction confirms FCC lattice and the measured inter-atomic d-spacing between the (200) plane is 2.73 Å. Thus, the obtained lattice constant is about 5.46 Å, which matches well with the refined Hf-MPO-1500-large lattice constant (Fig. 1d). The acquired low magnification STEM-EDS (Fig. 4b) from the same region of Fig. 4a displays the oxygen concentration of 67.59%, which can be assigned to Hf-MPO-1500-large. Further, from the STEM-EDS mapping (Fig. 4b), the oxygen aggregation with Hf is clearly evident. This observation clarifies Hf diffusion during annealing to form “oxygen-rich” and “oxygen-poor” regions even within a particle. Figure 4c,d HRTEM and STEM-EDS show the particle surface with the oxygen-poor region. Especially in the yellow squared region of Fig. 4d, the oxygen content is hardly seen from the oxygen mapping. From the thin edge of a particle (Fig. 4d), HRTEM image taken along the [110] direction is shown in Fig. 4c. The measured d-spacing value from the image (Fig. 4c) between (200) lattice planes is 2.616 Å and the corresponding estimated lattice constant is 5.232 Å. Compared to the oxygen-rich region, the lattice constant of this oxygen-poor region is significantly less, which corresponds to the characteristic of the Hf-MPO-1500-small phase. This result indicates that the existence of an oxygen-rich and oxygen-poor region in Hf-MPO-1500 causes peak splitting as evident from the XRD (Fig. 1). Meanwhile, HRTEM images (Fig. 4a,c) show sharp surface steps/kinks, which are more commonly found in many particles suggesting that this step surfaces may assist in releasing oxygen to generate vacancies in MPOs.

**Figure 5:**
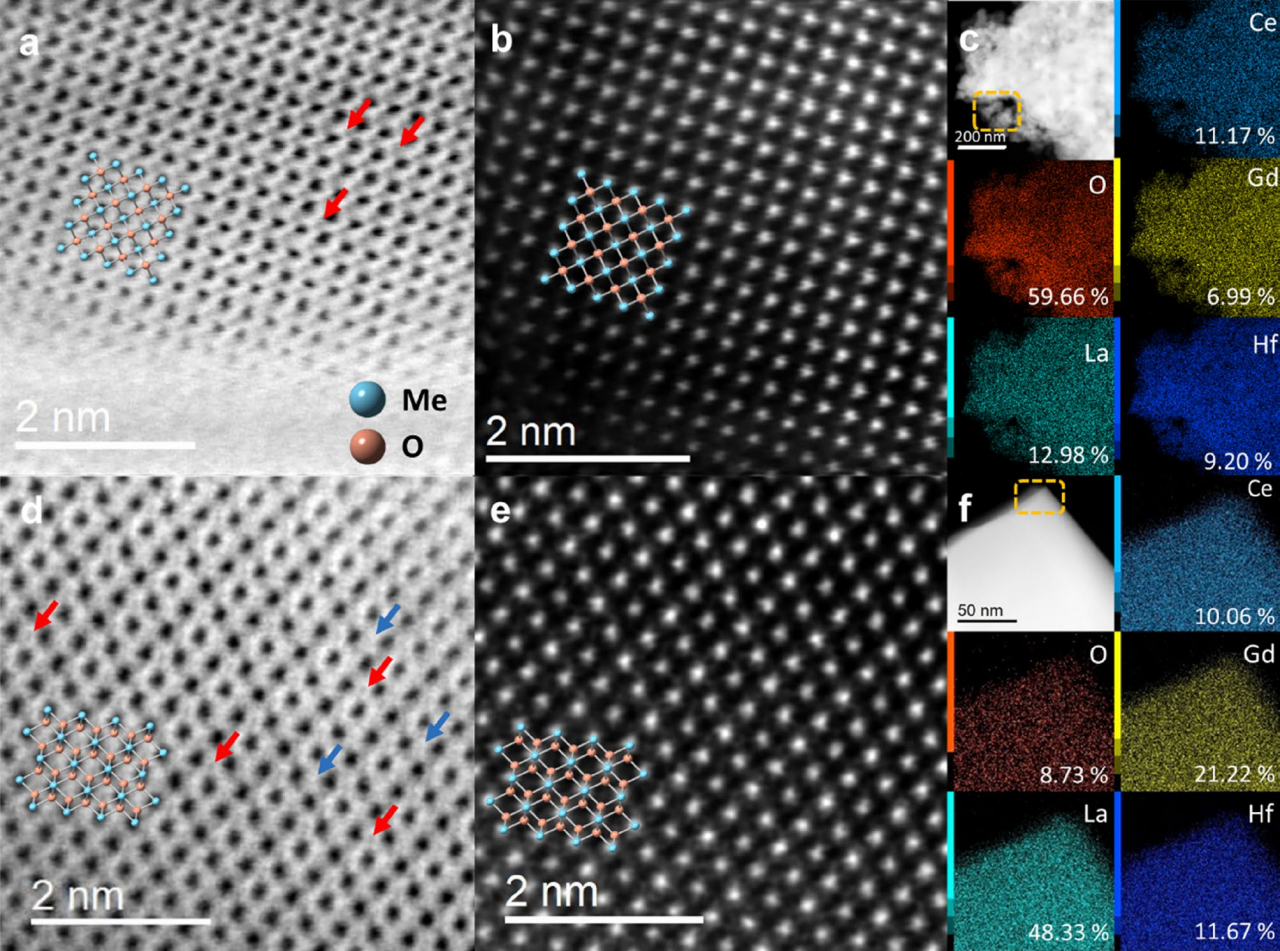
ABF-STEM, HADDF-STEM images and corresponding STEM-EDS results of (**a**–**c**) Hf-MPO-1000 along [100] direction and (**d**–**f**) oxygen-lack region in Hf-MPO-1500 along [110] direction. Red arrows pointed out the weak contrast of oxygen’s atomic column. Blue arrows pointed out the possible sites with a high concentration of oxygen vacancies.

To investigate the atomic structure of oxygen-poor and oxygen-rich regions, the high-resolution ABF-STEM and HAADF-STEM together with STEM-EDS were performed. Figure 5a,b shows the variation of oxygen occupancy in Hf-MPO-1500-small compared with Hf-MPO-1000. The STEM-EDS results of Hf-MPO-1000 (Fig. 5c) combined with the corresponding elemental content analysis show a homogeneous distribution except for some visual inhomogeneous distribution of oxygen due to the difference in thickness. To locate the oxygen atomic sites, an ABF-STEM image viewed along [100] direction on this Hf-MPO-1000 is shown in Fig. 5a. Apart from the bright-dark contrast of metal cations, the weak-dark contrast can be recognized from the center of four cations relating to O columns in Fig. 5b. Corresponding projected atomic model along [100] direction is overlapped on the ABF-STEM image to show the occupation site of cations and oxygen anions, which indicates a typical fluorite structure. While in the HADDF-STEM image, the intensity of an atom column is proportional to Z^n^ in which Z is the atomic number of the corresponding element in that column^22^, and n is 1.6–1.9 in most cases^23^. A high-resolution HADDF-STEM image viewed along [100] direction is shown in Fig. 5b to confirm if there is any nanoscale chemical short-range order. From this observation, it is clear that bright- and dark atoms are homogeneously distributed in the whole viewed regions, chemical short-range order is not obvious in Hf-MPO-1000 like reported in the literature^24^. However, the oxygen occupation was found to be different in Hf-MPO-1500-small phases. STEM-EDS mapping results with the elemental content analysis confirm the low oxygen contents with a homogeneous distribution of all 5 elements in this local region (Fig. 5e,f). Further, ABF-STEM observation along [110] direction is shown in Fig. 5d to reveal the oxygen occupation difference in the oxygen-poor region. The ideal fluorite model is overlapped on the ABF-STEM image to show the theoretical oxygen sites along [110] projection. Although the oxygen concentration is even lower than 10%, they still predominately visible as pointed by red arrows from this projection because the sample image (Fig. 5d) is acquired from whole atomic columns. Nevertheless, when the oxygen content in columns is low enough, the contrast appears with very low or even invisible (which is pointed out by blue arrows to show some oxygen columns with very weak contrast). The difference in oxygen columns visibility of Hf-MPO-1000 and Hf-MPO-1500-small directly shows the liberation of oxygen atoms during high temperature annealing process. Although high concentration of oxygen atoms diffused out of the lattice during heating process as evidenced from Fig. 5b, but the sublattice of metal cations still remains as FCC structure. Thus, we believe that this phenomenon could help Hf-MPO to be a good oxygen storage material with enhanced performance and good stability at a high operation temperature.

### Oxygen vacancies and phase separation effect on energy band gap transition

**Figure 6:**
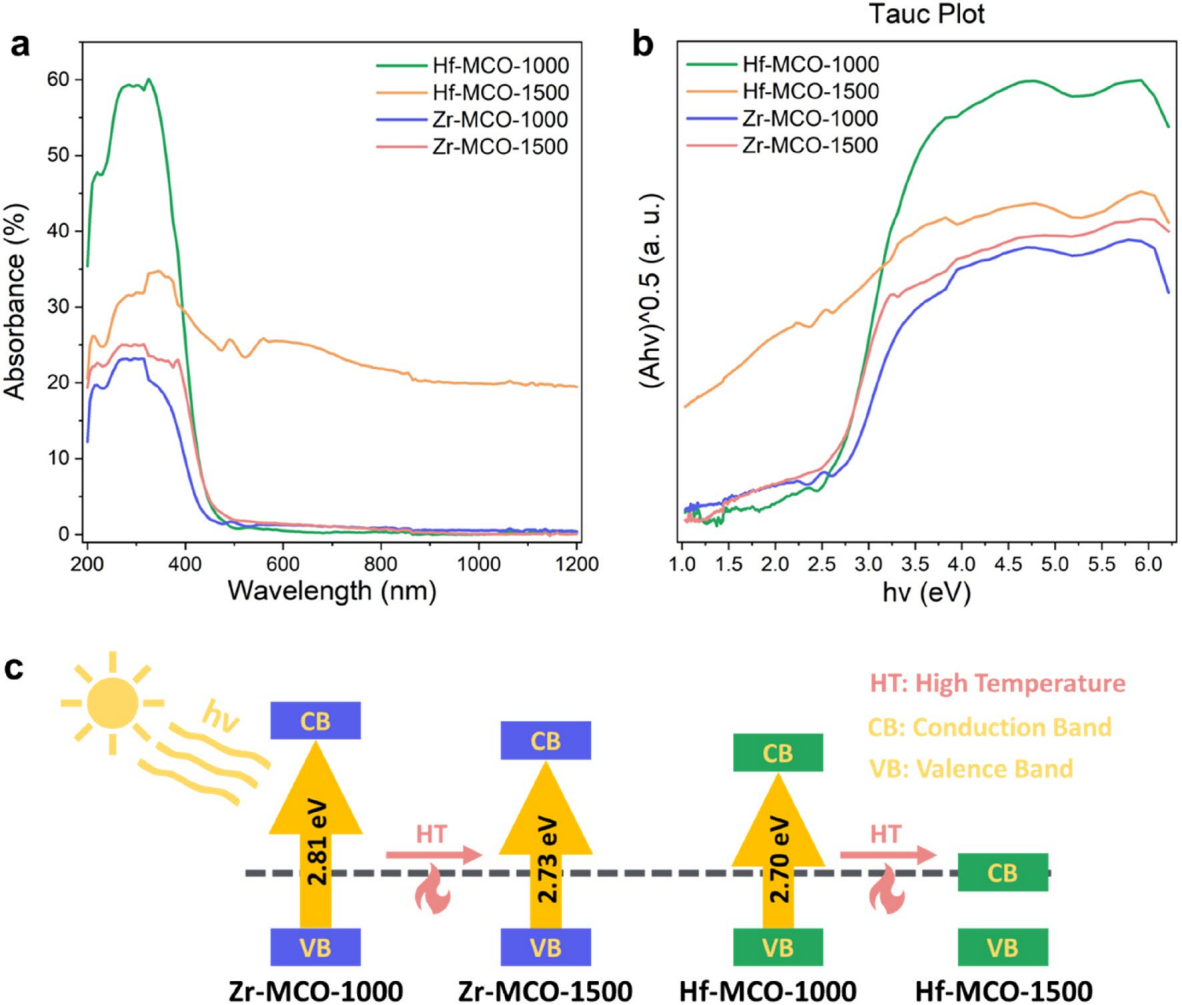
UV–vis spectra (**a**) and related Tauc Plot (**b**) of Hf-MPO-1000, Hf-MPO-1500, Zr-MPO-1000 and Zr-MPO-1500. (**c**) Schematic illustration of bandgap decreases by temperature-induced oxygen vacancies.

A fundamental understanding of how microstructural evolution affects macroscopic properties has been one of the most critical issues in materials science. Moreover, the oxygen vacancies in oxide semiconductors are proven to narrow the bandgap efficiently^25,26^. Thus, we adopted UV–Visible (UV–Vis) spectroscopy to study the overall band gap of the annealed MPOs and the obtained absorption spectrum and related Tauc plot are shown in Fig. 6a,b. As no band structures were reported before for this kind of MPOs, here the bandgap type of CeO_2_^27^, which is indirect, was adopted to generate the Tauc plot. Thus, the n factor in Tauc plot equation^28^ is determined to be 0.5. From UV–Vis spectroscopy measurement, it is clear that except Hf-MPO-1500 all the other MPOs in this study exhibit semiconductor features. The absorption edge appeared at a wavelength close to 450 nm and the light above this wavelength is transparent for these three MPOs, while there is no clear absorption edge for the UV–Vis spectroscopy of Hf-MPO-1500. From the Tauc plot in Fig. 6b, Hf-MPO-1500 exhibits a zero bandgap feature while the other 3 MPOs owned a band gap close to 2.7 eV (the determination method^29^ of band gap from Tauc Plot is shown in Fig. S4). The results of the semiconductor-to-metal energy transition in Fig. 6 reveal the electronic structure change in Hf-MPO-1500, in which OVG underwent more intense than in the other 3 MPOs. This transition feature is consistent with the identified metal-like MPO phase of oxygen-poor regions in Hf-MPO-1500, where oxygen vacancies are most abundant, and oxygen vacancies usually lead to conduction/valence band covering the fermi level^30^. In addition to oxygen-poor regions, the existence of oxygen-rich regions in the same particle of Hf-MPO makes the UV–vis spectrum and Tauc plot difficult to distinguish like in other oxides^29^. While in the case of Zr-MPO-1500, the generated oxygen vacancies are 12 at% higher compared to the Zr-MPO-1000 reflected in the 0.08 eV bandgap reduction (illustrated in Fig. 6c). The narrowed bandgap in Zr-MPO made the absorption edge in Zr-MPO red-shifted 26 nm, which widened the absorption area in the visible light region and made it more efficient as a potential photocatalyst.

### Effect of generated oxygen vacancies on photocatalytic performance

When the energy of phonons is higher than the band gap of a semiconductor oxide, electrons can be excited from the valence band to the conduction band, which can be used for further redox reactions. But the recombination of the photo-induced electron–hole pair can happen in a few nanoseconds, which stops the transfer of excited electrons for the redox reactions. As the generated oxygen vacancies act as active sites for improving the photocatalytic performance, they can also trap electrons, which increases the recombination rate and thus worsens the photocatalytic performance. To determine the recombination rate of the photogenerated charge carriers in the annealed Zr/Hf-MPOs, we have conducted photoluminescence (PL) studies (Fig. S5a). As all PL spectra show similar peak position and shapes, the relative intensity can be the indicator of photo-induced carrier recombination rate. The higher the PL signal, the higher the photo-induced carrier recombination rate^31^. It was found that the OVG indeed resulted in a higher PL signal in Zr-MPO after annealing at 1500 °C, indicating the higher recombination rate of photo-induced carriers. But a lower PL intensity is observed when more oxygen vacancies appear in Hf-MPO at 1500 °C. This follows the trend observed in other oxides: the PL intensity increases and then decreases with the increase in the number of oxygen vacancies^32^. Under the same heat treatment condition, Hf-MPO and Zr-MPO showed different OVG behavior, indicating the strong composition dependence of the OVG process. Further, the photocatalytic performance does not follow the same trend of photo-induced carrier recombination rate as shown in Fig. S5b. Generally, the Zr/Hf-MPO annealed at 1000 °C showed a better photocatalytic performance because of the smaller particle size, while Hf-MPO-1000 outperformed Zr-MPO-1000 because of the wider visible light absorbance. The annealed Zr/Hf-MPOs at 1500 °C showed poor photocatalytic performance due to the particle coarsening. These observations suggest that a balance between the oxygen vacancies and particle size in MPOs is crucial for enhanced photocatalytic properties.

## Discussion

Conventional metal oxides have been intensely studied to incorporate oxygen vacancies into the lattice to narrow the band gap for higher visible light utilization, but the structure degrades heavily with the introduction of oxygen vacancies^20^. For example, TiO_2_ degrades to Ti_2_O_3_ with oxygen loss, as the oxygen loss does not act as oxygen vacancies in the original lattice but convert from a tetragonal to a trigonal crystal structure^33^. Multi-principal materials (MPMs) with high entropy are generally considered to be more stable with increasing temperature^34^, this led to MPM applied in critical high temperature conditions^35,36^. According to the classical Gibbs–Helmholtz equation^37^, high entropy leads to a more significant decrease in Gibbs free energy with increasing temperature, which implies higher structural stability. But due to the complexity of MPMs, it sometimes possesses some unexpected properties^38,39^. Our results show that Hf-MPO is contrary to the high temperature stability based on the Gibbs–Helmholtz equation, while Zr-MPO is macroscopically stable at elevated temperatures. However, in the case of Hf-MPO and Zr-MPO, the huge oxygen loss brought by high temperature didn’t result in change of crystal structure, which remained as fluorite structure even in Hf-MPO-1500-small with very low oxygen contents. This proved the higher oxygen vacancy accommodation capacity in MPOs than in conventional metal oxide photocatalysts. The generated oxygen vacancies in MPOs bring extra configuration entropy to the existing system, which further lowered the Gibbs free energy at elevated temperature and compensated the energy penalty such as extra lattice distortion induced by existing oxygen vacancies. The more oxygen vacancies in MPOs can provide more accessible active sites for photocatalytic reactions, which promises a bright future for MPOs as potential photocatalysts.

Previously MPOs have been found to have potential applications as photocatalysts^10,11,13^ but the wide band gaps found in most MPOs^15^ limited the exploration toward practical photocatalysts. The modification of band gaps in MPOs is all achieved by composition adjustment^10,11,13^, mostly elemental replacement. There is a lack of a general method for MPOs to achieve band gap engineering. The OVG at elevated temperature found in Hf-MPO and Zr-MPO has proved the potential of being a possible solution for the common wide band gaps in MPOs. Our DFT calculations (Table S2) revealed higher oxygen affinity for Hf as compared to Zr (− 3.931 eV vs − 3.745 eV) causing different regions of grains with different oxygen capacities. With a feasible annealing process, the MPOs undergo marginal decrease in band gap to 0.08 eV in Zr-MPO or 0 band gap transitions in Hf-MPO. This proved the large tunability of oxygen vacancy concentration and electronic structures in MPOs. With proper control of annealing parameters, Hf-MPO can even transform from a wide band gap oxide semiconductor to metal-like phases with 0 band gap, which was never observed so far. Moreover, the OVG process can be easily tuned by adjusting composition, temperature and annealing time to generate oxygen vacancies to a different extent, which can lead to the band gap reduction to a desired extent. Thus, we believe that the controllable OVG process can help MPO materials with more advanced applications such as oxide electronics and electrocatalysts.

## Conclusion

In summary, the combined experimental observations and theoretical prediction revealed the temperature-induced OVG process in Zr/Hf-MPO. It is found that OVG is highly dependent on composition and temperature and resulted in a stable single phase in Zr-MPO but phase separation in Hf-MPO after annealing to 1500 °C. The phase separation in Hf-MPO-1500 was validated as two cubic fluorite phases with different lattice constants, which is the direct consequence of the different degree of OVG generated in these two phases. The difference in the degree of OVG in Hf-MPO-1500 stems from its composition-dependent nature and was triggered by the segregation of Hf aggravated by OVG at elevated temperatures. Further, the band gap measurements showed the great potential for bandgap engineering that the microscale OVG process can cause macroscale electronic structure change with a band gap reduction of 0.08 eV in Zr-MPO-1500 and semiconductor–metal transition in Hf-MPO-1500. Our findings of OVG in MPOs provide a general and feasible way for defects control and band gap engineering in MPOs.

## Methods

### Synthesis

The MPOs viz. (CeGdLaHf)O_x_ (Hf-MPO) and (CeGdLaZr)O_x_ (Zr-MPO) with nominal compositions were synthesized based on co-precipitation-peptization method^12,40,41^. For a typical Hf-MPO synthesis, 0.001 mol each of cerium ammonium nitrate (Ce(NH_4_)_2_(NO_3_)_6_, Sigma-Aldrich, 98.5%), gadolinium nitrate hydrate (Gd(NO_3_)_3_⋅H_2_O, Alfa Aesar, 99.9%), lanthanum nitrate hexahydrate (La(NO_3_)_3_⋅6H_2_O, Alfa Aesar, 99.9%) and hafnium chloride (HfCl_4_, Alfa Aesar, 98%) were firstly dissolved in a beaker filled with 30 ml deionized (DI) water. Similarly, for Zr-MPO synthesis, 0.001 mol of each cerium ammonium nitrate (Ce(NH_4_)_2_(NO_3_)_6_, Sigma-Aldrich, 98.5%), gadolinium nitrate hydrate (Gd(NO_3_)_3_⋅H_2_O, Alfa Aesar, 99.9%), lanthanum nitrate hexahydrate (La(NO_3_)_3_⋅6H_2_O, Alfa Aesar, 99.9%) and zirconyl chloride octahydrate (ZrOCl_2_⋅8H_2_O, Sigma-Aldrich, 98%) were dissolved in 30 ml DI water. Ammonia solution (NH_4_OH, Merck, 25%) was added into both the mixed solutions to increase the pH to above 12 in order to obtain the precipitates. The precipitates were then centrifuged and DI water was used to wash the precipitates several times until the final pH reached 7. Then 5 ml of DI water combined with nitric acid (HNO_3_, Merck, 69%) (1:1.5 metal ion to HNO_3_ ratio) were mixed with the washed precipitates and shaken vigorously. The obtained suspension was sonicated by a probe sonicator (Sonics Vibra-Cell VCX 750, 750 watts, 20 kHz) with an in-built temperature sensor. To prevent the solvent evaporation, a temperature threshold was set at 80 °C, which means the sonication stopped when the temperature of the solution reached 80 °C and started again when the temperature was lower than 80 °C. The whole sonication process lasted for about 1.5 h until a final transparent sol was obtained. The obtained sols were then dried in an oven for 24 h at 100 °C. Later, the dried powders of Hf-MPO and Zr-MPO were both calcined in a muffle furnace under an air atmosphere for 24 h at 1000 °C and another batch for 12 h at 1500 °C to study their behavior under a high temperature environment. The dried powders of Hf-MPO and Zr-MPO annealed at 1000 °C referred to as Hf-MPO-1000 and Zr-MPO-1000 while the MPOs annealed at 1500 °C referred to as Hf-MPO-1500 and Zr-MPO-1500, respectively.

### Material characterization

XRD analysis was performed in a multi-functional high-intensity 2-Dimensional (2D) X-ray Diffraction system (RIGAKU RINT RAPID II with MicroMax 007HF Cu/Cr Dual-target Rotating-anode). Material Analysis Using Diffraction (MAUD^®^)^42^ software was used to perform the Rietveld refinement of the XRD patterns and obtain the lattice constants. The atomic models were generated by CrystalMaker^®^ software. The morphology of MPOs was studied by scanning electron microscope (SEM, VERSA 3D, FEI). The DSC/TGA test was carried out with a Simultaneous Thermal Analyzer (STA) STA 449 F3 (NETSCZH GmbH). The sample was first kept in a muffle furnace at 300 °C under an air atmosphere for 4 h to drive out the moisture content and then sealed in a corundum pan for further DSC/TGA test. The sample was heated from 40 to 1500 °C under an air atmosphere at a ramp rate of 10 K/min. Raman-spectra were obtained using Renishaw inVia Qontor with a laser wavelength of 532 nm and laser beam spot of 1 µm. Two analytical transmission electron microscopes (JEOL ARM 200F) were used to characterize the microstructures of Hf-MPO and Zr-MPO. One of them, equipped with a cold field emission gun and an aberration corrector for the probe-forming lens system was used to obtain the atomic resolution STEM images. The other one, equipped with an aberration corrector for the objective lens system was used to obtain high-quality HRTEM images. Both TEMs were operated at 200 kV. High-angle annular dark-field scanning TEM (HAADF-STEM) images were recorded using an annular-type detector with a collection semi-angle of ∼100–269 mrad. STEM-EDX elemental maps were also acquired to reveal the chemical composition and phase distribution. For TEM sample preparation, the MPO powders were dispersed in ethanol under sonication for 3 min and then dropped on a copper grid with carbon membranes on it. Subsequently, the copper grid was dried under an oven lamp before loading onto a double-tilt TEM holder for structural characterization. The photoluminescence spectra were recorded using the FLS1000 Edinburgh Analytical Instrument. The photocatalytic performance was performed under a 500 Watt Xenon lamp.

### Band gap measurements

UV Visible spectra of the Hf-MPO and Zr-MPO powders were collected from 200 to 1200 nm using a UV–Vis-NIR spectrophotometer (PerkinElmer Lambda 950). The band gap energy was estimated using Tauc relation^20^:

$${[F({R}_{\infty })\cdot hv]}^{1/n}=A\cdot (hv-{E}_{g})$$

where $F({R}_{\infty })$ is the Kubelka–Munk function, $v$ is the photon’s frequency, $h$ is the Planck constant, ${E}_{g}$ is the band gap energy and A is a constant. The n factor is determined by the nature of electron transition and is 1/2 or 2 for direct and indirect band gaps, respectively. Linear regression was fitted on the curves of the Tauc plot (${[F({R}_{\infty })\cdot hv]}^{1/n}$ vs.$hv$) after the inflection point and the $hv$-intercept values were taken as band gap energies.

### Theoretical calculations

Vienna Ab initio Simulation Package (VASP)^43^ based on Density Functional Theory (DFT) was used to perform the first principle study of thermodynamic and electrical properties of the MPOs. The projector augmented wave (PAW)^44^ approach was adopted to describe the Ion–Electron interactions. Perdew-Burke-Ernzerhof (PBE) version of generalized gradient approximation (GGA)^45^ was used to describe the Exchange-Correlation potentials. Special Quasi-random Structure (SQS) approach^46^ offered by Alloy Theoretic Automated Toolkit (ATAT) code^47^ was adopted to simulate the random order of occupied 4 kinds of metal cations. In this study, a conventional FCC unit cell was used, as shown in Fig. 1b, which contains 4 metallic atoms at the face-centered sites and 8 O atoms (or O vacancies) at 8 tetrahedral interstices. Three kinds of supercells based on the unit cell 1 * 1 * 2 (112), 2 * 2 * 1 (221), and 2 * 2 * 2 (222) with 24, 48 and 96 atoms (including the O vacancies), respectively were used to ensure the accuracy of this calculation and reduce the errors caused by different random structures. The cutoff energy of the plane wave basis was set to be 520 eV for all 3 kinds of supercells. Monkhorst–Pack scheme^48^ was adopted to sample the Brillouin zone (BZ) with a K mesh of 5 * 5 * 2, 2 * 2 * 5 and 2 * 2 * 2 for supercells 112, 221 and 222, respectively. Convergence criteria for total energy and atomic force were 1 * 10^–6^ eV and 3 * 10^–2^ eV/Å, respectively, for all 3 supercells. The Birch-Murnaghan equation of state^49^ was used to fit the volume-energy curve and obtain the optimized lattice constants which were further used to build the lattice constants-oxygen contents relationship.

## Supplementary Information

Supplementary Information.

## Data Availability

All of the data supporting this work will be made available from the corresponding author upon reasonable request.
