# Supplementary material for: Effective band gap engineering in multi-principal oxides (CeGdLa-Zr/Hf)Ox by temperature-induced oxygen vacancies
Source: Sci Rep. 2023 Feb 9;13:2362. doi: 10.1038/s41598-023-29477-0 (PMC9911753; doi:10.1038/s41598-023-29477-0)
Supplement: Supplementary file 1 — Supplementary Information. [file 41598_2023_29477_MOESM1_ESM.docx]

Effective band gap engineering in multi-principal oxides (CeGdLa-Zr/Hf)O_x_ by temperature-induced oxygen vacancies

*Yixuan Hu^1^, Mariappan Anandkumar^2^, Joydip Joardar^3^, Xiaodong Wang^1^, Atul Suresh Deshpande^2^, Kolan Madhav Reddy^1^**

*^1^State Key Laboratory for Metal Matrix Composites, School of Materials Science and Engineering, Shanghai Jiao Tong University, Shanghai, 200240, China*

*^2^Department of Materials Science and Metallurgical Engineering, Indian Institute of Technology Hyderabad, Sangareddy-502285, India*

*^3^International Advanced Research Centre for Powder Metallurgy and New Materials (ARCI), Balapur P.O., Hyderabad 500005, Telangana, India*

*Correspondence Email: [kmreddy@sjtu.edu.cn](mailto:kmreddy@sjtu.edu.cn)

**Supplementary Material contains**

**Text 1 Surface morphology of MPOs after annealing.**

**Fig. S1 Rietveld refinement results** **of MPOs**

**Fig. S2 SEM images of MPOs after heat treatment.**

**Fig. S3 Summarized STEM-EDS of MPOs.**

**Fig. S4 Determination of band gap for MPOs.**

**Fig. S5 Photolumensescence and photocatalysis of MPOs.**

**Table S1 Basic information of individual binary oxides involved in Hf-MPO and Zr-MPO**

**Table S2 O affinity of the cations involved in this study**

**Text 1 Surface morphology of MPOs after annealing.**

SEM was carried out to characterize the morphology of both Zr-MPO and Hf-MPO samples after post-annealing at 1000 °C and 1500 °C as shown in Fig. S2. SEM images taken from Zr-MPO-1000 (Fig. S2a) and Hf-MPO-1000 (Fig. S2b) samples show similar morphologies consisting of nanoparticles with approximately 100 nm in size, which is consistent with the as-synthesized nanoparticles reported in the previous works^1, 2, 3^. At 1500 °C annealing temperature, the obvious grain growth was observed in both Hf-MPO-1500 and Zr-MPO-1500 samples as shown in Fig. S2c and S2d. Unlike loosely distributed nanoparticles in Hf-MPO-1000 and Zr-MPO-1000, the large agglomerates are evident in both Hf-MPO-1500 and Zr-MPO-1500 composing of tightly bound grain sizes in between 0.5 to 2 μm. Except for these large agglomerates with extensive grain growth (see Fig. S2c and S2d), no other morphology changes were observed in Hf-MPO-1500 suggesting the phase separation phenomenon revealed from XRD spectra (Fig. 1) could have associated with inherent structural decomposition that is invisible from the surface mophology observation.

**
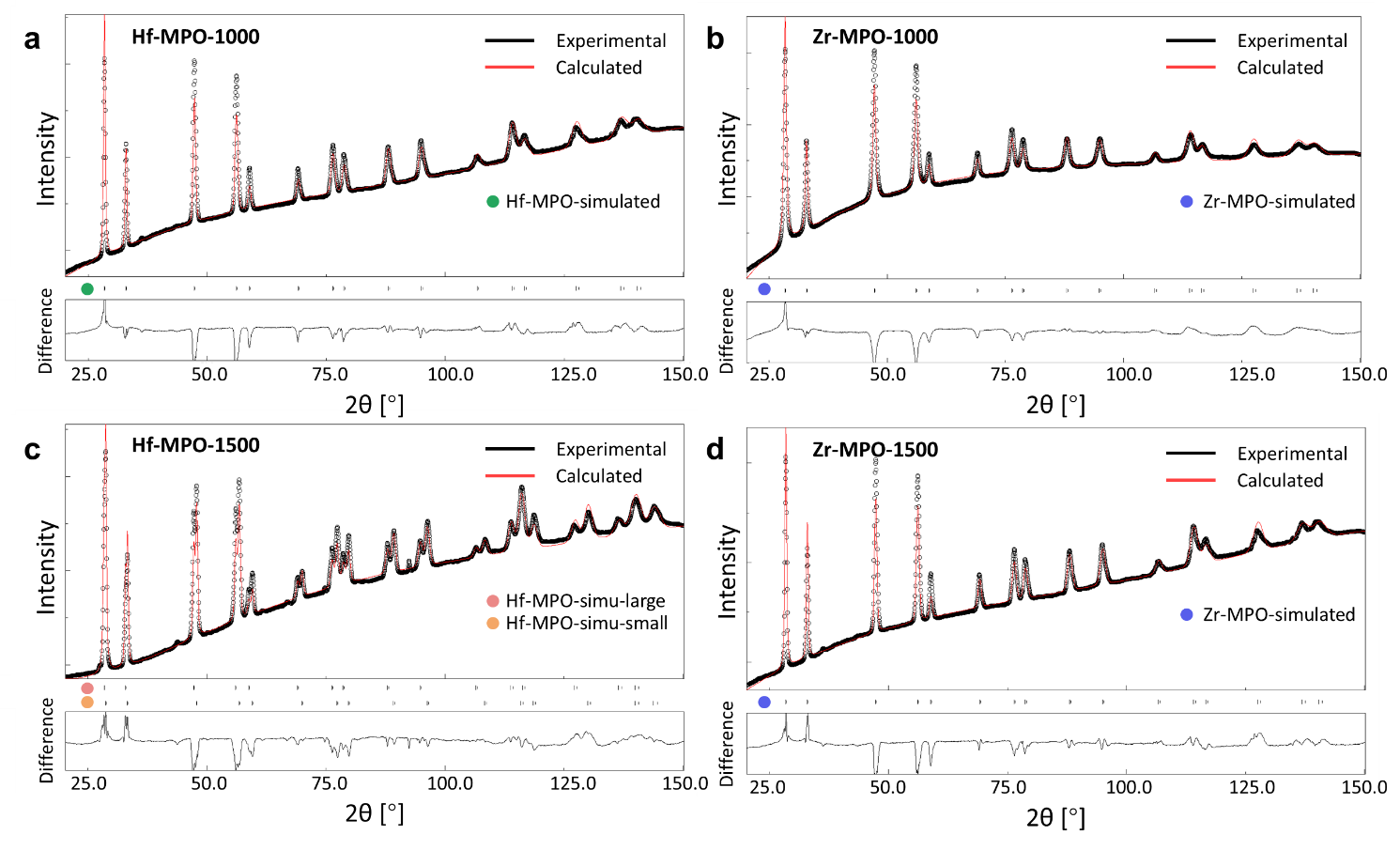
**

**Fig. S1** **Rietveld refinement results of MPOs.** (**a**) Hf-MPO-1000, (**b**) Zr-MPO-1000, (**c**) dual phase Hf-MPO-1500, (**d**) Zr-MPO-1500. Error function Rb values are 3.3043 %, 3.6891 %, 3.2553 %, 3.0891 % for Hf-MPO-1000, Hf-MPO-1500, Zr-MPO-1000, Zr-MPO-1500 respectively.


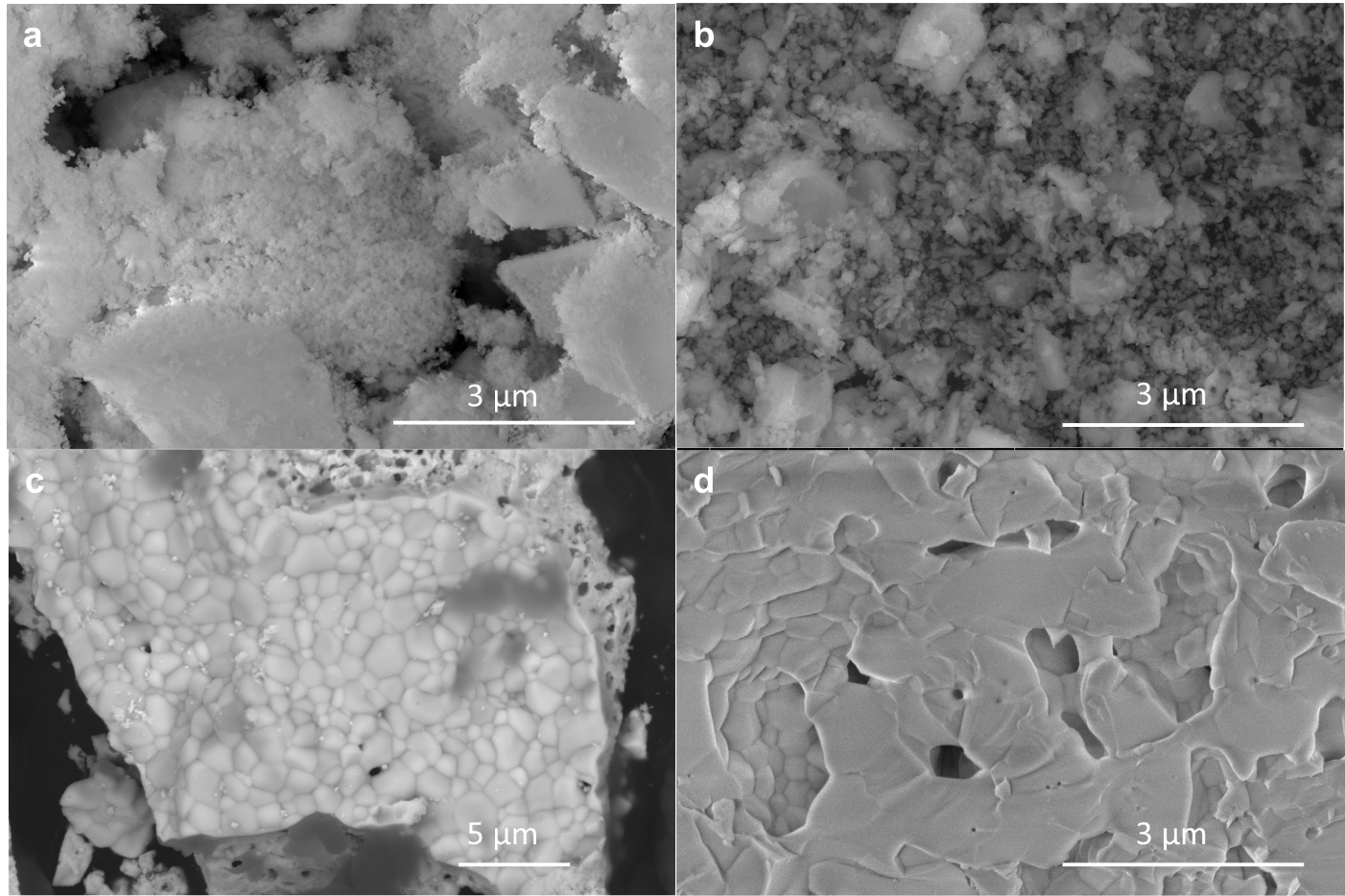


**Fig. S2 SEM images of MPOs after heat treatment.** (a) Hf-MPO-1000; (b) Zr-MPO-1000; (c) Hf-MPO-1500; (d) Zr-MPO-1500.


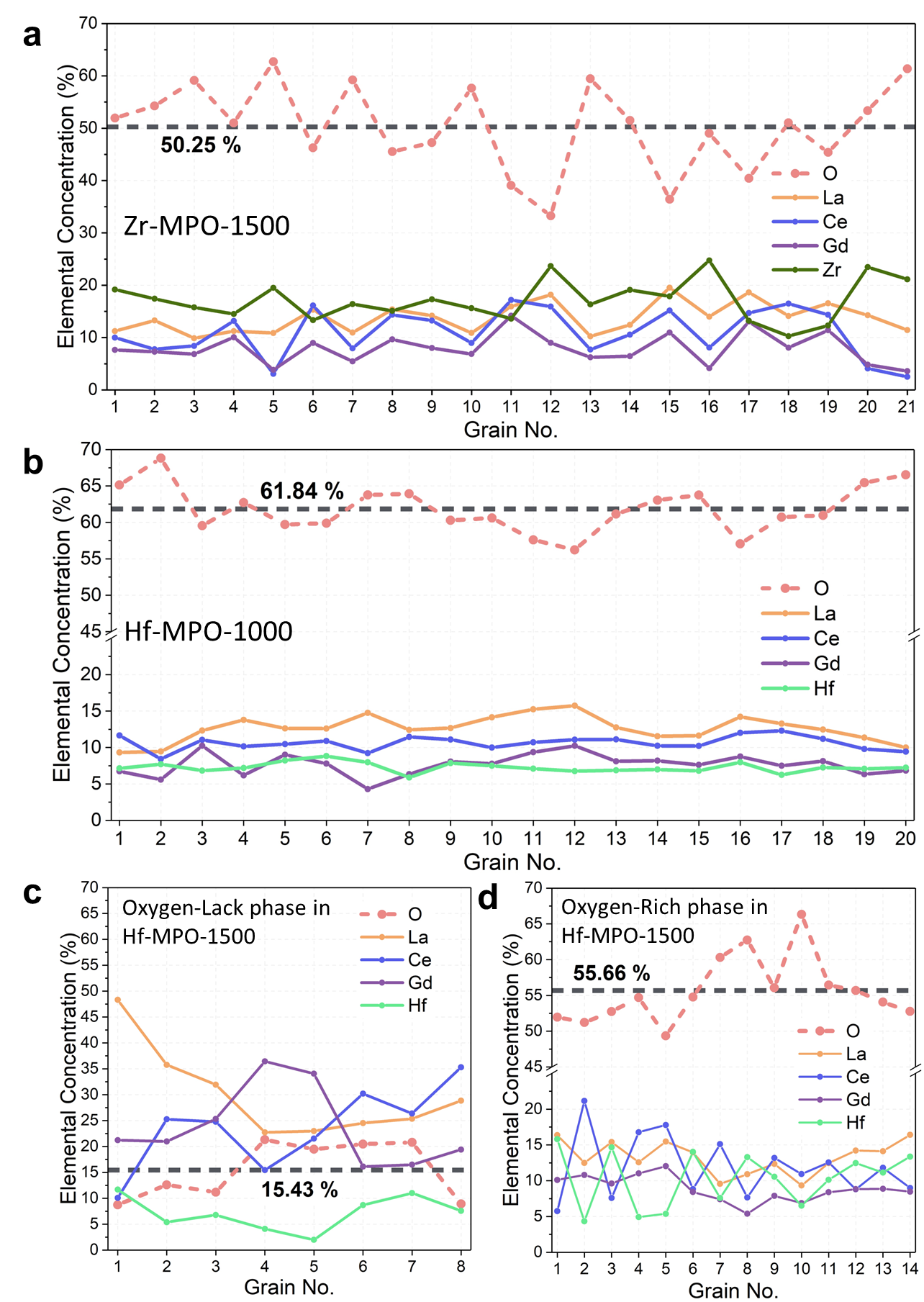


**Fig. S3 Summarized STEM-EDS of MPOs.** Elemental analysis results in different regions of (**a**) Hf-MPO-1000; (**b**) Zr-MPO-1500; (**c**) Oxygen-lack phase in Hf-MPO-1500; (**d**) Oxygen-rich phase in Hf-MPO-1500. The dotted black line points out the average oxygen concentration of these phases.


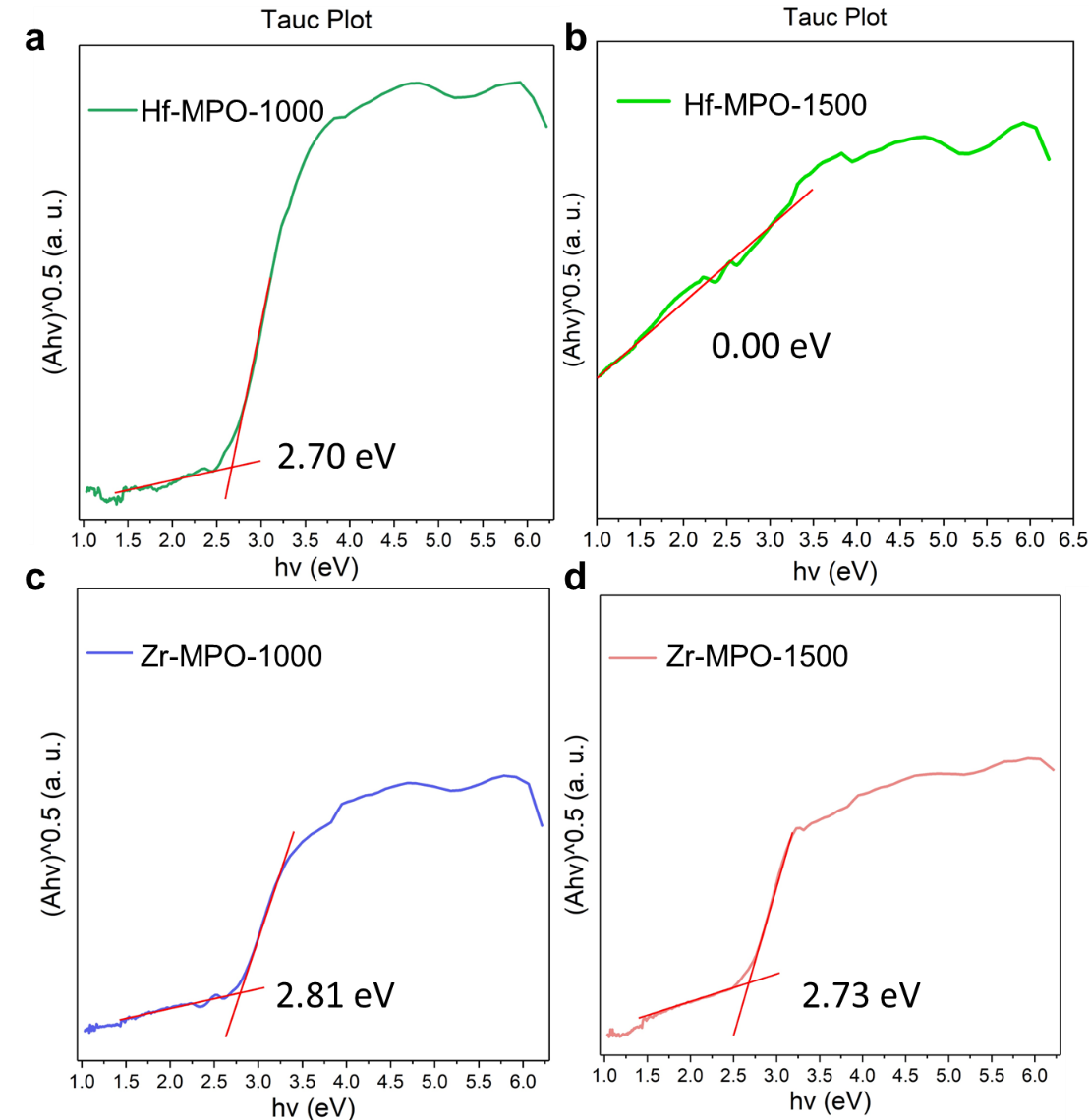


**Fig. S4 Determination of band gap for MPOs.** (**a**) Hf-MPO-1000, (**b**) Hf-MPO-1500, (**c**) Zr-MPO-1000 and (**c**) Zr-MPO-1500.


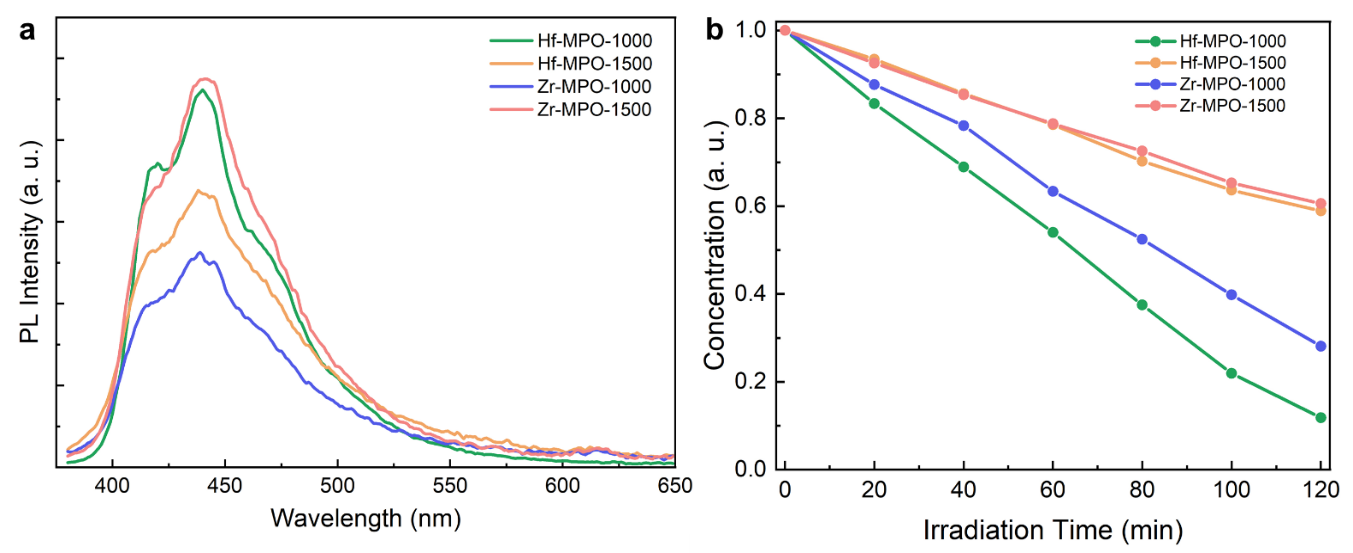


**Fig. S5 Photolumensescence and photocatalysis of MPOs.** (a) Photoluminescence spectra of annealed Zr/Hf-MPOs; (b) Performance of photocatalytic reduction of Cr(VI) utilizing Zr/Hf-MPOs as photocatalysts.

**Table S1 Basic information of individual binary oxides involved in Hf-MPO and Zr-MPO**

|  | Melting Point (℃) | Boiling Point (℃) | Cation Valence State | Ionic Radius | oxide structure |
| --- | --- | --- | --- | --- | --- |
| ZrO_2_ | 2,715 | 4,300 | +4 | 0.86 | monoclinic, dopant stabilized cubic fluorite |
| HfO_2_ | 2,758 | 5,400 | +4 | 0.85 | monoclinic, dopant stabilized cubic fluorite |
| CeO_2_ | 2,400 | 3,500 | +4, +3 | 1.15 | cubic fluorite |
| Gd_2_O_3_ | 2,420 | N/A | +3 | 1.08 | Cubic |
| La_2_O_3_ | 2,315 | 4,200 | +3 | 1.36 | Hexagonal |

**Table S2 O affinity of the cations involved in this study**

| Cation | Formation energy of related fluorite structure |
| --- | --- |
| Ce^4+^ | -3.928 eV/atom |
| Gd^3+^ | None |
| La^3+^ | None |
| Hf^4+^ | -3.931 eV/atom |
| Zr^4+^ | -3.745 eV/atom |

The affinity of a certain cation is represented by the formation energy of the related fluorite structure such as CeO_2_ and HfO_2_. The Gd^3+^ and La^3+^ are not listed as they don’t exist in fluorite structure stably.

**References**

1. Anandkumar M, Bhattacharya S, Deshpande AS. Low temperature synthesis and characterization of single phase multi-component fluorite oxide nanoparticle sols. *RSC Advances* **9**, 26825-26830 (2019).

2. Anandkumar M, Bagul PM, Deshpande AS. Structural and luminescent properties of Eu3+ doped multi-principal component Ce_0.2_Gd_0.2_Hf_0.2_La_0.2_Zr_0.2_O_2_ nanoparticles. *Journal of Alloys and Compounds* **838**, (2020).

3. Anandkumar M, Lathe A, Palve AM, Deshpande AS. Single-phase Gd_0.2_La_0.2_Ce_0.2_Hf_0.2_Zr_0.2_O_2_ and Gd_0.2_La_0.2_Y_0.2_Hf_0.2_Zr_0.2_O_2_ nanoparticles as efficient photocatalysts for the reduction of Cr(VI) and degradation of methylene blue dye. *Journal of Alloys and Compounds* **850**, (2021).
